# Long Chain Fatty Acyl-CoA Synthetase 4 Is a Biomarker for and Mediator of Hormone Resistance in Human Breast Cancer

**DOI:** 10.1371/journal.pone.0077060

**Published:** 2013-10-14

**Authors:** Xinyu Wu, Yirong Li, Jinhua Wang, Xin Wen, Max T. Marcus, Garrett Daniels, David Y. Zhang, Fei Ye, Ling Hang Wang, Xinxin Du, Sylvia Adams, Baljit Singh, Jiri Zavadil, Peng Lee, Marie E. Monaco

**Affiliations:** 1 Department of Neuroscience & Physiology, New York University School of Medicine, New York, New York, United States of America; 2 Department of Pathology, New York University School of Medicine, New York, New York, United States of America; 3 Department of Pediatrics, New York University School of Medicine, New York, New York, United States of America; 4 Department of Medicine, New York University School of Medicine, New York, New York, United States of America; 5 NYU Cancer Institute, New York University School of Medicine, New York, New York, United States of America; 6 NYU Center for Health Informatics and Bioinformatics, New York University School of Medicine, New York, New York, United States of America; 7 Department of Pathology, Mount Sinai School of Medicine, New York, New York, United States of America; 8 VA New York Harbor Healthcare System, New York, New York, United States of America; Sun Yat-sen University Medical School, China

## Abstract

The purpose of this study was to determine the role of long-chain fatty acyl-CoA synthetase 4 (ACSL4) in breast cancer. Public databases were utilized to analyze the relationship between ACSL4 mRNA expression and the presence of steroid hormone and human epidermal growth factor receptor 2 (HER2) in both breast cancer cell lines and tissue samples. In addition, cell lines were utilized to assess the consequences of either increased or decreased levels of ACSL4 expression. Proliferation, migration, anchorage-independent growth and apoptosis were used as biological end points. Effects on mRNA expression and signal transduction pathways were also monitored. A meta-analysis of public gene expression databases indicated that ACSL4 expression is positively correlated with a unique subtype of triple negative breast cancer (TNBC), characterized by the absence of androgen receptor (AR) and therefore referred to as quadruple negative breast cancer (QNBC). Results of experiments in breast cancer cell lines suggest that simultaneous expression of ACSL4 and a receptor is associated with hormone resistance. Forced expression of ACSL4 in ACSL4-negative, estrogen receptor α (ER)-positive MCF-7 cells resulted in increased growth, invasion and anchorage independent growth, as well as a loss of dependence on estrogen that was accompanied by a reduction in the levels of steroid hormone receptors. Sensitivity to tamoxifen, triacsin C and etoposide was also attenuated. Similarly, when HER2-positive, ACSL4-negative, SKBr3 breast cancer cells were induced to express ACSL4, the proliferation rate increased and the apoptotic effect of lapatinib was reduced. The growth stimulatory effect of ACSL4 expression was also observed *in vivo* in nude mice when MCF-7 control and ACSL4-expressing cells were utilized to induce tumors. Our data strongly suggest that ACSL4 can serve as both a biomarker for, and mediator of, an aggressive breast cancer phenotype.

## Introduction

Breast cancer is a heterogeneous disease comprised of distinct molecular subtypes that can be generally characterized by the expression status of receptors for estrogen (ER), progesterone (PR), human epidermal growth factor receptor 2 (HER2) and more recently, androgen (AR). Tumors that are negative for expression of ER and PR and for amplification of HER2 expression are termed triple negative breast cancers (TNBC) and display a more aggressive phenotype that is not amenable to steroid hormone/HER2-based targeted therapies and has a worse prognosis than receptor positive cancer [1]. Recent studies indicate that TNBC can be further stratified on the basis of expression of AR [2-4]. TNBC lacking AR are considered quadruple negative breast cancers (QNBC) and have been demonstrated to have a worse prognosis than TNBC in most studies [5], though not all [6]. 

We have previously demonstrated that the fatty acid metabolic enzyme, long chain fatty acyl-CoA synthetase 4 (ACSL4) is differentially expressed in human breast cancer samples as a function of expression of ER and AR [7]. ACSL4 is one of five isoforms of mammalian long chain acyl-CoA synthetases that activates fatty acids for further metabolism by condensing the fatty acid with a molecule of coenzyme A to form a thioester. Individual ACSL isoforms are characterized by their subcellular location and substrate specificity, although the significance of these characteristics has yet to be fully elucidated [8]. ACSL4 is unique in that it is localized to the peroxisome and the mitochondria-associated endoplasmic reticulum membrane, and has higher affinity for arachidonic acid (AA) and eicosapentanoic acid as substrates [9]. Previous studies have demonstrated that ACSL4 is overexpressed in both liver [10] and colon [11] cancer as well as in aggressive forms of breast cancer [7,12], and it has been suggested that metabolism of AA may play a role in mediating the effects of ACSL4 expression [12,13]. We showed that in both breast cancer cell lines and tumor samples, ACSL4 expression is inversely correlated with ER and AR levels. Importantly, in ER-negative tumors, high ACSL4 expression predicts a shorter time to distant metastases [7]. Thus ACSL4 serves as one of many biomarkers of an aggressive breast cancer phenotype and/or resistance to hormonal interventions. These data raise the question of the function of ACSL4 enzyme activity in mediating the aggressive phenotype associated with hormone independence. The investigation of the role of ACSL4 enzyme activity in mediating the aggressive phenotype associated with hormone independence may aid in the discovery of new therapeutic targets.

In the current study, we expand our previous findings to include a negative correlation between ACSL4 expression and HER2 amplification, and determine that ACSL4 levels correlate positively with the most aggressive QNBC. Furthermore, we demonstrate the impact of induced ACSL4 expression on cell growth, invasion and resistance to hormones. 

## Materials and Methods

### Ethics Statement

 Commercially available human cell lines and anonymous patient data from public databases were used for this study; as such, no patient consents were required and an exemption was granted by the Subcommittee for Human Studies of the Veterans’ Administration Medical Center on 12/09/08. Approval for animal experiments was obtained from the Institutional Animal Care and Use Committee of NYU School of Medicine (NYU IACUC # 110506-02).

### Tissue culture, cell proliferation and invasion assays

MDA-MB-231 cells, MCF-7 cells and SKBr3 cells (American Type Culture Collection) were maintained in DMEM (Life Technologies) supplemented with 10% heat-inactivated bovine serum (fetal bovine serum), 1 U/ml of penicillin and 1 μg/ml of streptomycin. Cell proliferation was measured by the colorimetric WST assay under different growth conditions, in either complete medium, or phenol red-free medium containing charcoal-stripped with or without defined levels of estrogen (1 nM 17β- estradiol). Anchorage-independent cell growth in soft agar was performed in triplicate with cells (4x10^4^) suspended in 2ml of medium containing 0.35% agar (Becton Dickinson) spread on top of 5ml of 0.7% solidified agar. Numbers and size of colonies were calculated.

Matrigel invasion assays were performed by adding 750 μL media with a chemoattractant (5% FBS) to the lower chamber of a BD Biocoat Matrigel Invasion Chamber (BD Bioscience, Bedford). A cell suspension (5 x 10^4^) in 0.5 ml DMEM with 0.1% BSA was placed on the insert of the 24-well chamber. After 18 hours of incubation, the non-invading cells on the upper surface of the filter member were removed with a cotton swab. Invasive cells on the lower surface of the filter member were stained via Diff Quik stain and counted under light microscopy. The percentage of invasion was expressed as the ratio of invading cells over cell number normalized on day 2 of the growth curve. These methods have been previously described [14].

### Transfection of MCF-7 and SKBr3 cells

To establish a stable cell line that conditionally expressed ACSL4, the MCF-7 Tet-On Advanced Cell Line (Clonetech, CAT# 631153), grown in DMEM medium supplemented with 400 μg/ml G418, was subsequently transfected with either the pTRE-ACSL4 plasmid or the empty pTRE plasmid as a control. Individual cell clones were selected and isolated in the presence of 400 μg/ml G418 and 2 μg/ml puromycin in the culture medium. Single cell clones were cultured at high cell density in the presence and absence of 1 μg/ml doxycycline for 24 h and ACSL4 expression confirmed by immunoblot analyses.

A second method was utilized to force expression of ACSL4 in MCF-7 and SKBr3 cells. The Precision LentiORF-ACSL4 and RFP control viral particles (Thermo Scientific Company, CAT # OHS5833 and OHS5899) were incubated with MCF-7 and SKBr3 cells for 15 hours in high glucose DMEM containing 10% FBS, 10 μg/ml polybrene (Sigma) at 37°C in an atmosphere of 5% CO_2_. On the next day, the infected cells were washed twice with DMEM medium and individual cell clones were selected and grown in the presence of 10 μg/ml Blasticidin S. 

### RNA extraction, semi-quantitative RT-PCR and siRNA knockdown

Total RNA was extracted from cells using the RNAqueous-4PCR kit (AM1914, Ambion). RetroScript kit (AM1710, Ambion) was used for cDNA synthesis with isolated RNA as template, according to manufacturer’s instructions. 5 μl of the reverse transcription mixtures was used as template in 50 μl reactions. The PCR parameters were set as follow: 95°C -30 sec, 60°C-30 sec and 72°C- 30 sec. 15 μl of PCR product was separated on 2% agarose gel. 

Small Interfering RNA-mediated knockdown of ACSL4 in MDA-MB-231 cells was performed in T-25 flasks in complete medium lacking antibiotic after cells were allowed to attach overnight. Cell densities at the start of the experiment were between 30% and 60%. Transfection of small interfering RNA (siRNA; either control or ACSL4-specific Smart Pool siRNA purchased from Dharmacon, Lafayette, CO) was accomplished using Lipofectamine RNAiMAX (Invitrogen) according to the protocol recommended by the manufacturer. Transfections were carried out for 48 hours.

### Analysis of microarray data

Gene expression in MCF-7 and cells was analyzed with Affymetrix GeneChip Arrays. Three individual biological replicate samples of RNA were assayed for each experimental condition. The target populations and GeneChips were prepared, hybridized, and scanned according to the manufacturer's instruction. Briefly, 1 μg total RNA, isolated as described above, was reverse transcribed with a poly-(T) primer containing a T7 promoter, and the cDNA made double-stranded. An in vitro transcription was done to produce biotinylated cRNA, which was then hybridized to the GeneChips. The chips were washed and stained with streptavidin-conjugated phycoerythrin using an Affymetrix FS-450 fluidics station, and data was collected with Affymetrix GeneChip Scanner 3000. 

 Microarray expression data was processed by Robust multichip average (RMA) normalization by GenePattern in the ExpressionFileCreator module and/or GeneSpring GX11 software (Agilent). CEL files were transformed into GCT format with normalized probe set intensity values. Comparative Marker Selection was used to calculate p-value, FDR, FWER, and fold change to select statistical significant candidate probe sets. Gene Set Enrichment Analysis (GSEA) and NIH DAVID databases were used to interpret the ranked probe sets to identify significantly enriched biological processes and gene-based categories in the Gene Ontology, KEGG pathway and Reactome databases. Microarray data has been deposited at the Gene Expression Omnibus web site (GSE40968). 

### Immunoblot analysis

Immunoblot analysis was used to assess expression of ACSL4 and ER, with β-actin utilized as a loading control. Methods were as previously described [15]. In brief, the cells were lysed in an appropriate volume of lysis buffer containing protease cocktail inhibitor (P8340, Sigma) and the extracts were separated using SDS-polyacrylamide gel electrophoresis. The proteins were transferred to a PVDF nitrocellulose membrane for western blot analysis and developed with antibodies against ACSL4 (S0101, Epitomics), ER (SC-542, santa cruz), and β-actin (A5441, Sigma), and with the appropriate horseradish peroxidase-conjugated secondary antibody (7076S or 7074S, cell signaling). Protein bands were identified by imaging with a ChemiDoc XRS system. Densities were quantitated using the Quantify One 4.6.9 software system (Biorad). 

### Apoptosis assays

Control- and ACSL4-transfected cells were treated with tamoxifen, triacsin C, etoposide or lapatinib in complete medium for 72 hours. Apoptosis was measured using the Caspase-Glo assay kit (Promega, Madison USA). Briefly, after the plates containing cells were equilibrated at room temperature for 30 minutes, 100 μl of Caspase-Glo reagent was added to each well, the content of the well was gently mixed with a plate shaker at 300–500 rpm for 30 seconds followed by incubation at room temperature for 8 hours. The luminescence value of each sample was measured with luminometer (Thermo Labsystems) using 1 minute lag time and 0.5 second/well read time. The experiments were performed in triplicate and repeated on two separately-initiated cultures. 

### Proteomic Pathway Array Analysis

The analyses were carried out as previously described [16-19]. In brief, proteins were extracted from cells using a lysis buffer containing 20 mmol/L Tris-HCl (pH 7.5), 20 mmol/L sodium pyrophosphate, 40 mmol/L B-glycerophosphate, 30 mmol/L sodium fluoride, 2 mmol/L EGTA, 10 mmol/L NaCl, and 0.5% NP-40. The lysate was sonicated 3 times for 15 seconds each time, and then centrifuged (14,000 rpm, 30 minutes, and 4°C). The tubes were kept on ice throughout the process. The protein concentration was determined with the BCA Protein Assay Kit (PIERCE). Isolated proteins were separated by SDS-PAGE (10% acrylamide). Three hundred micrograms of protein extracts were loaded in a well across the entire width of gel for SDS-PAGE, followed by electro-transfer to a nitrocellulose membrane. The membrane was then blocked for 1 hour with 5% milk or 3% bovine serum albumin and clamped on to a Mini-PROTEAN II Multiscreen apparatus that isolates 20 channels across the membrane (Bio-Rad). Two or 3 antibodies were added to each channel and incubated overnight at 4°C. Different sets of antibodies were used for each membrane after stripping the previous set of antibodies. Antibodies were purchased either from Cell Signaling Technology, Inc., or from Santa Cruz Biotechnology, Inc. Two separate analyses were run for each sample. In each set, antibodies and protein levels were normalized by using β-actin and glyceraldehyde-3-phosphate dehydrogenase as standards. Chemiluminescence signals were captured by using the ChemiDoc XRS System. Differences in protein levels were determined by densitometric scanning and normalized to internal standards.

### Xenograft studies in nude mice

Control-transfected and ACSL4-expressing MCF-7 cells were generated by stable transfection with lentiviral particles as described above. 1 x 10^6^ cells from a single clone were mixed with Matrigel (Becton Dickinson) at a ratio of 1:1 and inoculated into the right inguinal mammary gland of 4- to 5-week-old female Nu/Nu BALB/c athymic nude mice (Frederick National Laboratory, NCI Animal Program). Both intact and ovariectomized animals were evaluated. The animals were given no exogenous estrogen. There were 10 animals for each experimental condition. The tumor growth was monitored and tumor volume measured every 3 days. The tumor volume was calculated as l x d x h x 0.52 [20].

### Statistical Analyses

Data was analyzed using the two-tailed Student t-test to compare means and the 2-way ANOVA test to compare growth curves. Differences were considered statistically significant for p< 0.05. For calculation of the predicative value of ACSL4 as a biomarker, a diagnostic test evaluation was carried out as described (http://www.medcalc.org/calc/diagnostic_test.php). 

## Results

### QNBC express high levels of ACSL4

 Publicly available microarray data was analyzed to determine the correlation between ACSL4 mRNA expression and expression of steroid hormone/HER2 receptors (ER, PR, AR and HER2). Results consistently revealed an inverse relationship between ACSL4 expression and receptor status. Table 1 illustrates this finding for several different studies of either cell lines [21,22] or tumor samples [23-26]. When one compares ACSL4 mRNA expression levels in ER-positive with that in ER-negative cells, the p value is 8.0E-05. Separating the samples by TNBC status decreases this p value by 10-fold, and adding AR-negative samples to the TNBC cohort, referred to as quadruple negative breast cancer (QNBC), further increases the significance of the difference. Utilizing expression array data from a different experiment where a total of 51 cell lines were analyzed, 21 of which were not included in the first analysis, the results for differential expression of ACSL4 versus QNBC yielded a p value of 4.59E-08. Thus two separate experiments yielded the same results. 

**Table 1:** Differential expression of ACSL4 in breast cancer as a function of receptor status.

**Study**	**Sample Type**	**Target Group (no.)**	**Other Group (no.)**	**TG/OG**	**p value**
Neve [21]	Cell lines	ER- (31)	ER+ (19)	1.71	8.00E-05
		TNBC (24)	Other (26)	1.79	7.00E-06
		QNBC (22)	Other (28)	1.88	3.75E-08
Hoeflich [22]	Cell lines	QNBC (22)	Other (29)	12.29	4.59E-08
Hess [23]	Tumor	TNBC (58)	Other (120)	1.33	6.70E-03
TGCA^1^	Tumor	TNBC (49)	Other (300)	1.54	7.30E-05
Wang [25]	Tumor	TNBC (55)	Other (200)	1.57	2.55E-10
Waddell [26]	Tumor	TNBC (22)	Other (44)	2.24	4.08E-06

Data was taken from arrays published by the authors. The p value, calculated using a two-tailed Student t-test, is for the difference in ACSL4 expression values between the designated groups. TG/OG is the ratio of the relative ACSL4 value for the Target Group divided by the Other Group. The number in parentheses denotes the sample size.

1 Data deposited at www.oncomine.com

Results were similar in studies of tumor samples also shown in Table 1. In evaluating ACSL4 mRNA levels in tumor samples, contamination with stromal and normal tissue must be considered. To determine the potential relevance of such contamination in evaluating ACSL4 levels, we determined ACSL4 expression levels in normal and stromal tissue utilizing microarray data reported for microdissected breast tumor tissue [27]. Results indicate that stromal tissue expresses high levels of ACSL4, while normal tissue expresses moderate levels (data not shown). This is not surprising since neither stromal tissue nor the majority of epithelial cells in normal tissue expresses receptors [28]. Thus in evaluating the expression data for human tumor samples, contamination with stroma and normal tissue might explain the variability in results when compared with those seen for cloned breast cancer cell lines. Note also that the ratio of the relative ACSL4 values between the groups does not take into account that the lower relative values have been empirically determined to represent the absence of ACSL4 expression, as we have previously determined by immunoblot analysis [7]

### Predictive value of ACSL4 as a biomarker for QNBC

Tables 2 and 3 detail the ACSL4 and receptor status of 71 individual cell lines. The ACSL4 status was determined for the cell lines by comparing the relative expression values to those previously validated by immunoblot [7]. The status for ER, PR and HER2 were derived from the relevant publications [21,22]. The status for AR was determined from microarray data. Table 2 lists cell lines that are positive for one or more receptors, while table 3 lists those cell lines that are QNBC. A statistical analysis was carried out to determine the utility of ACSL4 status (positive or negative) as a predictor of QNBC status. Table 4 summarizes the statistical data regarding the relationship between ACSL4 expression and QNBC status in these cell lines. The estimated value for sensitivity is 78% and for specificity is 86%. The positive and negative predictive values were not calculated because prevalence of QNBC in the cell lines was not comparable to that seen in breast cancer specimens. Of particular interest is the subset of cells that either co-express ACSL4 and a receptor or fail to express either ACSL4 or a receptor. These cell lines are listed in table 5. While it is possible that these represent false positives or false negatives, it is also possible that these cells comprise separate molecular subtypes (ACSL4+, non-QNBC and ACSL4-, QNBC) with implications for prognosis and treatment response. Thus addition of ACSL4 status as a biomarker might increase the predictive value of receptor status alone, and allow us to define a new category of effective QNBC for those specimens that express both ACSL4 and receptors. Those QNBC that fail to express ACSL4 might be designated as pseudo-QNBC. 

**Table 2:** ACSL4 expression in steroid hormone/HER2 receptor positive breast cancer cell lines.

	**ACSL4**	**HER2**	**ER**	**PR**	**AR**
**Cell Line**					
600MPE	N	N	P	N	N
AU565	N	P	N	N	N
BT474	N	P	P	P	P
BT483	N	N	P	N	N
Cama1	N	N	P	N	P
EFM19	N	N	P	N	N
EFM192A	N	P	P	N	N
HCC1007	N	P	P	N	P
HCC1008	N	N	P	N	N
HCC1419	N	P	P	N	N
HCC1428	N	N	P	N	N
HCC1569	P	P	N	N	N
HCC1954	P	P	N	N	N
HCC202	N	P	N	N	P
HCC2218	N	P	N	N	P
KPL1	N	N	P	N	N
KPL4	N	P	P	N	P
LY2	N	N	P	N	N
MCF-7	N	N	P	P	N
MDA-134VI	N	N	P	N	P
MDA175VII	N	N	P	N	N
MDA361	N	N	P	P	N
MDA415	P	N	P	N	P
MDA453	N	N	N	N	P
MDA468	N	N	N	N	N
MFM223	N	N	N	N	P
SKBr3	N	P	N	N	N
SUM185PE	N	N	N	N	P
SUM190PT	P	P	N	N	N
SUM225CWN	N	P	N	N	N
SUM44PE	N	N	P	N	P
SUM52PE	N	N	P	N	N
T47D	N	N	P	P	N
UACC812	N	P	P	N	N
UACC893	N	P	N	N	P
ZR75-1	N	N	P	N	P
ZR75-30	N	P	P	N	P
ZR75B	P	N	P	N	N

Data for ER, PR, HER2 and AR were derived as described in the text from public databases. ACSL4 status was determined based the correlation between expression data and representative immunoblot data previously published [7]. P=positive; N=negative

**Table 3:** ACSL4 expression in steroid hormone/HER2 receptor negative breast cancer cell lines.

	**ACSL4**	**HER2**	**ER**	**PR**	**AR**
**Cell Line**					
BT-20	P	N	N	N	N
BT549	P	N	N	N	N
CAL120	P	N	N	N	N
CAL148	N	N	N	N	N
CAL51	N	N	N	N	N
CAL85-1	P	N	N	N	N
DU4475	P	N	N	N	N
EVSA-T	N	N	N	N	N
HCC1143	N	N	N	N	N
HCC1187	N	N	N	N	N
HCC1395	P	N	N	N	N
HCC1500	P	N	N	N	N
HCC1599	P	N	N	N	N
HCC1806	N	N	N	N	N
HCC1937	P	N	N	N	N
HCC2157	P	N	N	N	N
HCC2185	N	N	N	N	N
HCC3153	P	N	N	N	N
HCC38	P	N	N	N	N
HCC70	P	N	N	N	N
HDQ-P1	P	N	N	N	N
HS578T	P	N	N	N	N
JIMT1	P	N	N	N	N
MCF10A	P	N	N	N	N
MCF12A	P	N	N	N	N
MDA231	P	N	N	N	N
MDA436	P	N	N	N	N
MDAMB157	P	N	N	N	N
MX1	P	N	N	N	N
SUM1315	P	N	N	N	N
SUM149PT	P	N	N	N	N
SUM159PT	P	N	N	N	N
SW527	P	N	N	N	N

Source of data and abbreviations as described for Table 2.

**Table 4:** Predictive value of ACSL4 as a marker for QNBC in breast cancer cell lines.

	EstimatedValue	95% Confidence Interval	
		Lower Limit	Upper Limit
Sensitivity	0.787879	0.606013	0.903687
Specificity	0.868421	0.711162	0.950527
Positive Likelihood Ratio	5.99	4.38	8.19
Negative Likelihood Ratio	0.24	0.13	0.47

Data was derived using the ACSL4 status for the cell lines indicated in tables 2 and 3. In a total of 71 cell lines, 31 are QNBC and 40 are positive for one or more receptor. Of the QNBC cell lines, 26 are positive for ACSL4 and 5 are negative. Of the receptor-positive cell lines, 7 are positive for ACSL4 and 33 are negative.

Sensitivity: probability that ACSL4 will be positive when QNBC is present (true positive rate).

Specificity: probability that ACSL4 will be negative when QNBC is not present (true negative rate).

Positive likelihood ratio: ratio between the probability of a positive ACSL4 result given the presence of QNBC and the probability of a positive ACSL4 given the absence of QNBC, i.e.

= True positive rate / False positive rate = Sensitivity / (1Specificity)

Negative likelihood ratio: ratio between the probability of a negative ACSL4 result given the presence of QNBC and the probability of a negative ACSL4 result given the absence of QNBC, i.e.

= False negative rate / True negative rate = (1Sensitivity)/ Specificity

**Table 5:** Cell lines that are anomalies with respect to ACSL4 expression.

**Cell line**	**ACSL4**	**ER**	**PR**	**HER2**	**AR**
HCC1569	P	N	N	P	N
HCC1954	P	N	N	P	N
MDA415	P	P	N	N	P
SUM190PT	P	N	N	P	N
ZR75B	P	P	N	N	N
CAL148	N	N	N	N	N
CAL51	N	N	N	N	N
EVSA-T	N	N	N	N	N
HCC1143	N	N	N	N	N
HCC1187	N	N	N	N	N
HCC1806	N	N	N	N	N
HCC2185	N	N	N	N	N

Source of data and abbreviations as described for Table 2.

### Relationship of ACSL4 expression to intrinsic molecular subtype

**Figure 1:**
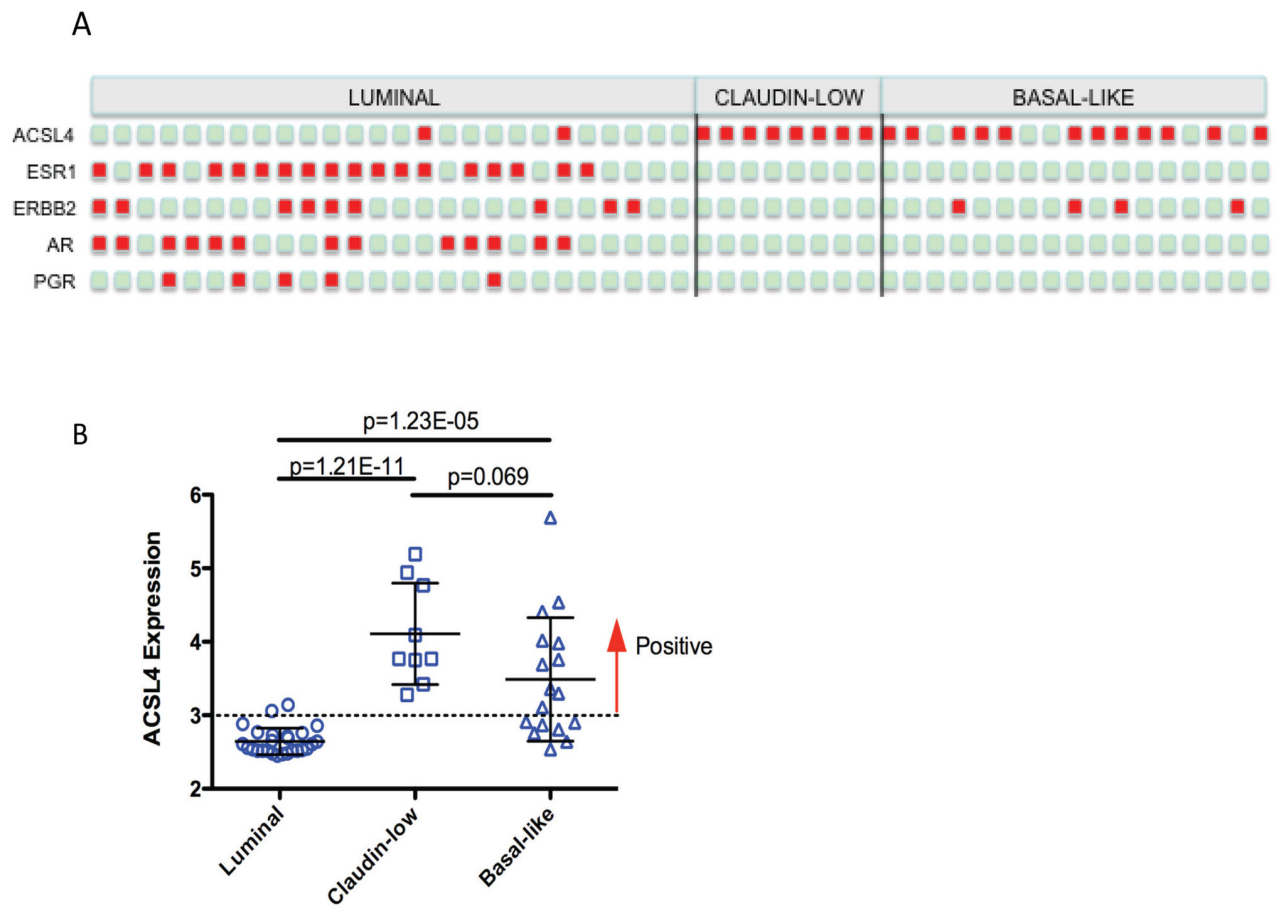
ACSL4 expression as a function of intrinsic molecular subtype. Microarray expression data and subtype allocations of 52 human breast cancer cell lines were obtained from previously published data [21,30]. Panel A lists expression status for ACSL4, ER (ESR1), HER2 (ERBB2), AR and PR (PGR). Green squares are negative for expression and red squares are positive. Identification of individual cell lines can be found in reference 30 (note: HBL-100, included in reference 30, was not included in this figure). Panel B shows ACSL4 mRNA relative expression values as a function of the molecular subtype of individual cell lines. Means and 1SD are shown, as well as the p values for the differences between subtypes.

 Although receptor status generally aligns with molecular subtype, that is not always the case [29]. For example, around 20% of TNBC are non-basal-like while 30% of basal-like tumors are non-TNBC. Using molecular subtype characterizations previously described for 52 breast cancer cell lines [30], as well as receptor status described by Neve et al [21] for the same cell lines, we examined ACSL4 status as a function of molecular subtype as shown in Figure 1. Figure 1A details the receptor status and ACSL4 status for each individual cell line, while 1B indicates the range, mean and standard deviation of ACSL4 values as a function of molecular subtype. 

 A similar correlation is seen with respect to ACSL4 mRNA expression in human tumor samples. In a series of tumor samples comprised of 5 classified as basal-like, 6 classified as ERBB2-enriched and 16 classified as luminal [22], ACSL4 mRNA expression differed among the subtypes as follows: luminal, 317 ± 130, basal-like, 565 ± 128, and ERBB2-enriched, 364 ± 83. The difference between ACSL4 mRNA expression in luminal and basal-like tumors was significant (p=0.001) as was that between ERBB2 and basal-like (p=0.011). There was no significant difference between expression in luminal and ERBB2-enriched (p=0.417). By contrast, although the trend was the same, there was no significant difference between the subtypes for expression of CD44, generally considered a marker for claudin-low and basal-like subtypes. 

### ACSL4 promotes growth of ER-positive breast cancer cells *in vitro*

**Figure 2:**
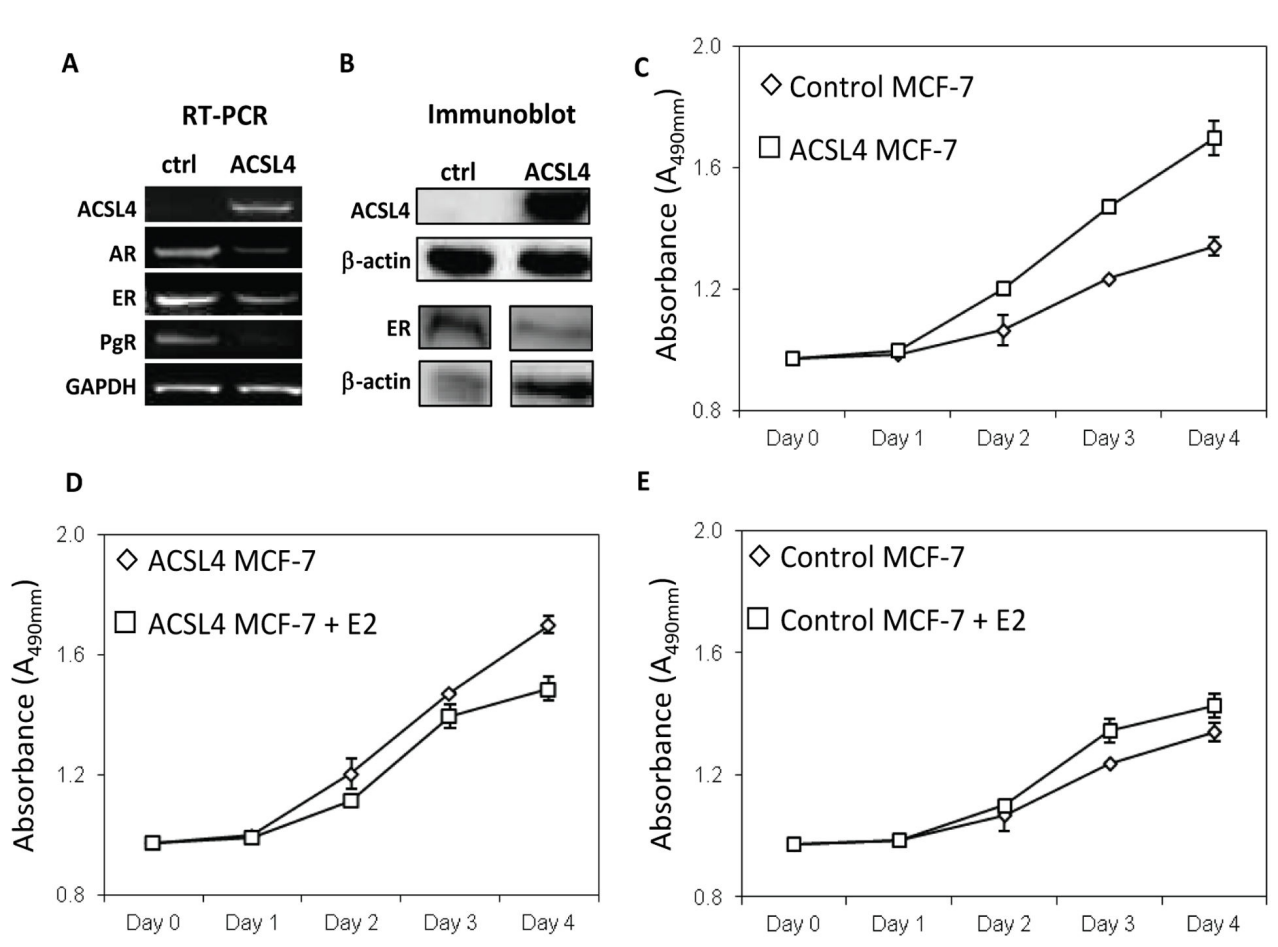
Effect of ACSL4 expression on breast cancer growth . A doxycycline-inducible line of MCF-7 cells was transfected with either a control or ACSL4-expressing plasmid as described in the text. Both control and ACSL4-MCF-7 cells were treated with 1μg/ml of doxycycline. (**A**) RT-PCR analysis of mRNA for ACSL4, AR, ER and PgR in vector control and ACSL4-MCF-7 cells. GAPDH was used as a loading control. (**B**) Immunoblot analysis of whole cell lysates showing expression of ACSL4 and ER with β-actin as loading control. The increase in ACSL4 is 163 fold, and the decrease in ER is 68%. (**C**) Comparison of proliferation of control with ACSL4-MCF-7 cells grown in phenol red-free medium supplemented with charcoal-stripped FBS. Values shown are the means of triplicate determinations ± 1SD. The difference between the curves is significant, p<0.0001. (**D**) The effect of estradiol on proliferation of doxycycline-induced ACSL4-transfected MCF-7 cells grown as in (**C**). Values shown are the means of triplicate determinations ± 1SD. The difference between the curves is significant, p<0.0001. (**E**) The effect of estradiol on proliferation of doxycycline-treated control MCF-7 cells grown as described above for panel **C**. Values shown are the means of triplicate determinations ± 1SD. The difference between the curves is significant, p<0.0001.

As we have previously demonstrated, neither ER-positive MCF-7 nor HER2-positive SKBr3 cells normally express ACSL4 as assessed by immunoblot [7]. Utilizing a doxycycline-dependent conditional expression system, the effect of ACSL4 expression on MCF-7 cells was examined. When compared with control cells, ACSL4-expressing MCF-7 cells exhibited an increased rate of growth (Figure 2C). In addition, ACSL4-expressing MCF-7 cells no longer responded to estradiol treatment with an increase in growth (Figure 2D), although an increase was seen in control-transfected MCF-7 cells (Figure 2E). In fact, a decrease was observed in the presence of estradiol. This loss of estrogen dependent growth was accompanied by a decreased expression of ER, PR and AR (Figure 2A and 2B).

**Figure 3:**
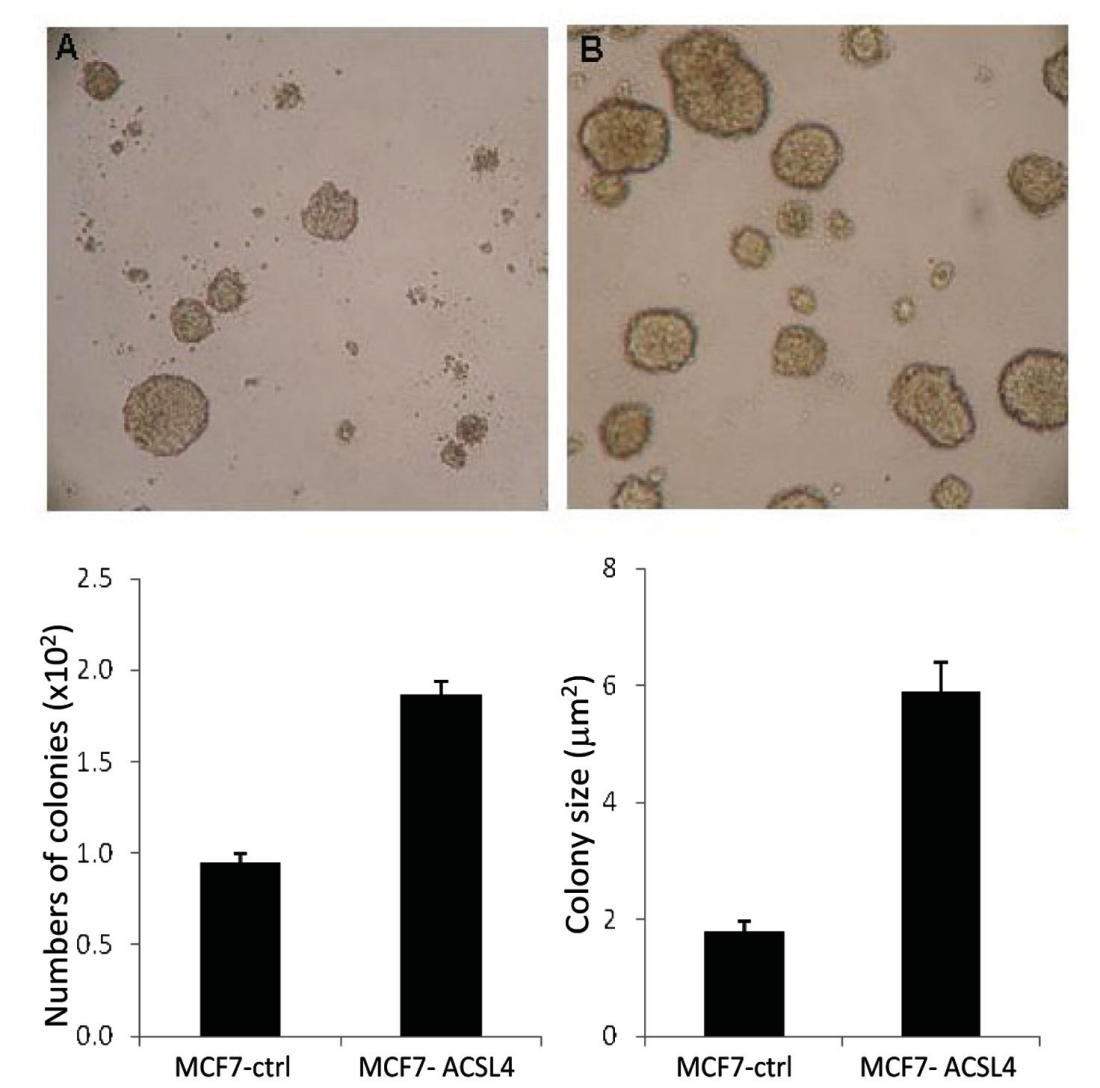
Effect of ACSL4 expression on anchorage-independent growth of MCF-7 cells. MCF-7 cells were stably transfected with ACSL4 cDNA utilizing a lentivirus vector, and control- and ACSL4-tranfected cells compared with respect to anchorage-independent growth as described in the text. Panel A,shows control cells, and panel B are ACSL-4-expressing cells. Panel C quantitates the number of colonies and panel D, the size of the colonies. Values shown in (C) and (D) are the means of triplicate determinations ± 1SD. The differences shown are significant: For C, p= 0.028 and for D, p=0.009.

**Figure 4:**
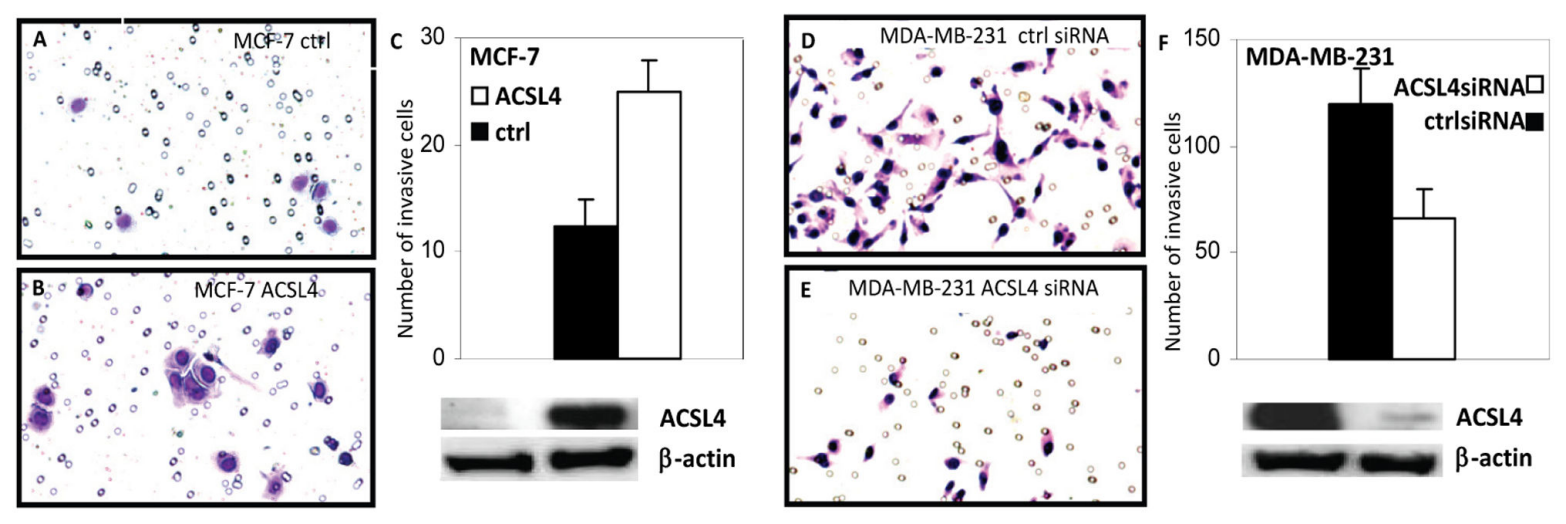
Effect of ACSL4 expression on invasion capability of breast cancer cells. MCF-7 cells were stably transfected with ACSL4 cDNA utilizing a lentivirus vector, and control- and ACSL4-tranfected cells compared with respect to invasive potential as described in the text. Panel **A**, control MCF-7, **B**, ACSL4-MCF-7, **C**, bar graph indicating the average number of cells per field in 3 separate chambers (p=0.005), and an immunoblot blot analysis of ACSL4 expression in control and ACSL4- MCF-7 cells. For panels **D**, **E** and **F**, MDA-MB-231 were treated with either control or ACSL4 siRNA (20nM), and control and experimental cells were compared with respect to invasive potential as described in the text. Panel **D**, control siRNA-treated cells, **E**, ACSL4 siRNA-treated cells, **F**, bar graph indicating the average number of cells per field in 3 separate chambers (p=0.005), and an immunoblot blot analysis of ACSL4 expression in control siRNA and ACSL4 siRNA-treated cells.

ACSL4 expression also increased anchorage-independent growth of MCF-7 cells. As demonstrated in Figure 3, both the number and size of the colonies increased significantly (Figure 3A and 3B). In addition to ACSL4’s effect on anchorage-independent growth of ACSL4-expressing MCF-7 cells, invasion capability, as measured by BD matrigel invasion assays, was also increased as shown in Figure 4 A-C. Consistent with this finding, when ACSL4-positive, QNBC MDA-MB-231 cells were treated with ACSL4 siRNA, as previously described [7], invasion capability was diminished (Figure 4 D-F).

**Figure 5:**
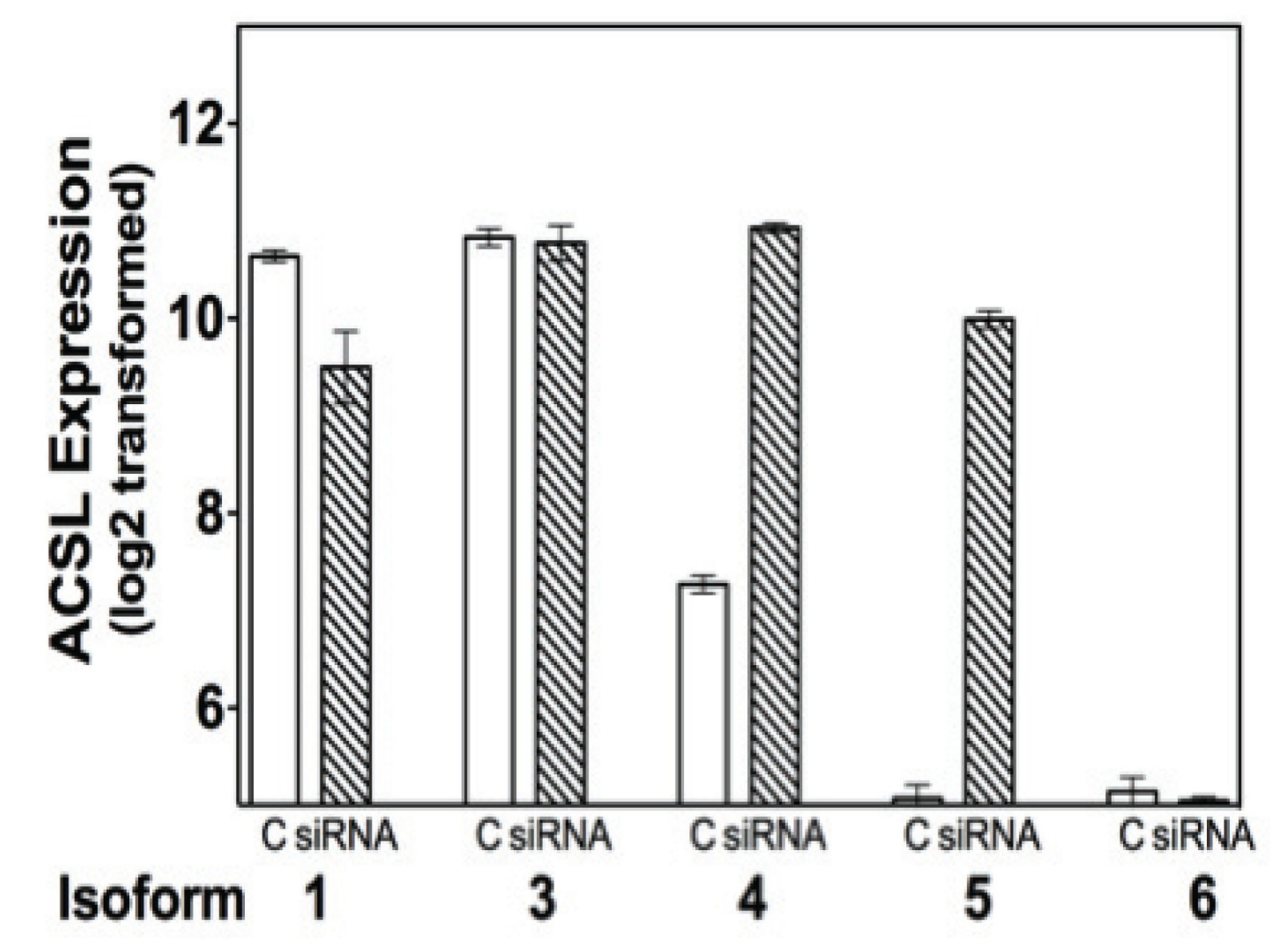
Effect of ablation of ERα on ACSL4 expression in MCF-7 cells. siRNA-was utilized to mediate the silencing of ER expression in MCF-7 cells as described in reference [31]. The results shown are taken from an Affymetrix Human Genome U133 plus 2.0 Gene Chip microarray study reported on the Gene Expression Omnibus (GDS40610). Values shown represent the means ± 1SD of triplicate determinations. The significance of the differences for isoform 1, p=4.53E-03, for isoform 4, p=5.02E-07, and for isoform 5, p=9.67E-07. No significant difference is observed for isoforms 3 and 6.

Since forced ACSL4 expression reduces expression of ER in MCF-7 cells, we speculated whether the reverse would be true, that is, whether reduction of ER would impact ACSL4 expression. Figure 5 details results derived from mRNA expression data as previously reported [31]. In this report the authors treated MCF-7 cells with siRNA directed against ER and subsequently carried out microarray studies to assess alterations in gene expression resulting from the decrease in ER. ACSL4 mRNA expression is increased as a result of the decrease in ER, while there is no significant effect on either ACSL3 or ACSL6. ACSL1 is decreased, while ACSL5 is also increased. The effects seen on ACSL1 and ACSL5 consequent to ER ablation are not observed when comparing ACSL expression in breast cancer cell lines as a function of ER expression, as we have previously reported [7]. However, an inverse relationship between ACSL5 and ER status was observed in our previous analysis of tumor sample data [7], although the results were not as significant as those we reported for ACSL4.

### ACSL4 enhances tumor growth of ER-positive breast cancer cells *in vivo*

**Figure 6:**
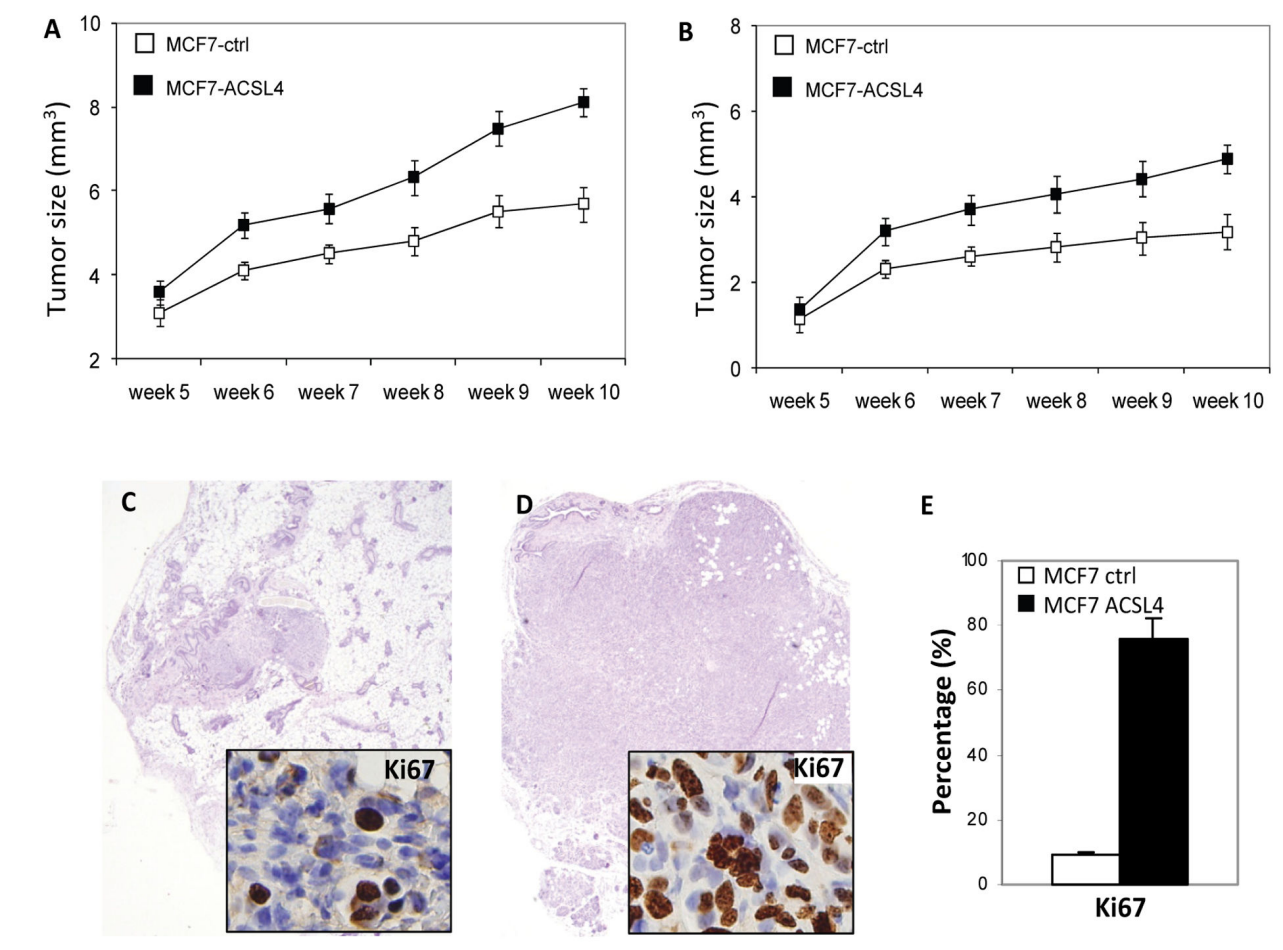
Comparison of the growth of control and ACSL4-MCF-7 cell tumors in nude mice. Nude mice, either intact (**A**) or ovariectomized (**B**) were injected with 1 × 10^7^ MCF-7 vector control- (open circles) or ACSL4-transfected (open squares) cells. Each group contained 10 animals. The differences between the curves were significant (**A**, p<0.0001 and **B**, p<0.0001). Panel **C** and **D** depict H and E stained samples from control and ACSL4-transfected tumors, respectively. Staining for Ki67 is shown in the inset of **C** and D. Panel **E** compares the percentage of cells in each sample that stain positively for Ki67. Three sections of each slide were analyzed to determine the significance of the difference (p=0.001).

To determine whether the growth regulatory effects of ACLS4 expression in MCF-7 cells could be observed *in vivo*, we performed orthotopic intramammary tumor xenograft experiments using intact and ovariectomized nude mice. The results shown in Figure 6 reveal there is an increased rate of tumor growth in xenografts overexpressing ACSL4 (n=10) compared to vector control (n=10) (p=0.0118) consistent with the data derived from cell culture experiments. This effect of ACSL4 expression on tumor proliferation was observed in both intact and ovariectomized mice. Immunohistochemical analysis of tumor samples indicated a significant increase in Ki67 staining in samples derived from ACSL4-expressing cells when compared to controls (Figure 6C-E).

### ACSL4 promotes proliferation of HER2-positive breast cancer cells

**Figure 7:**
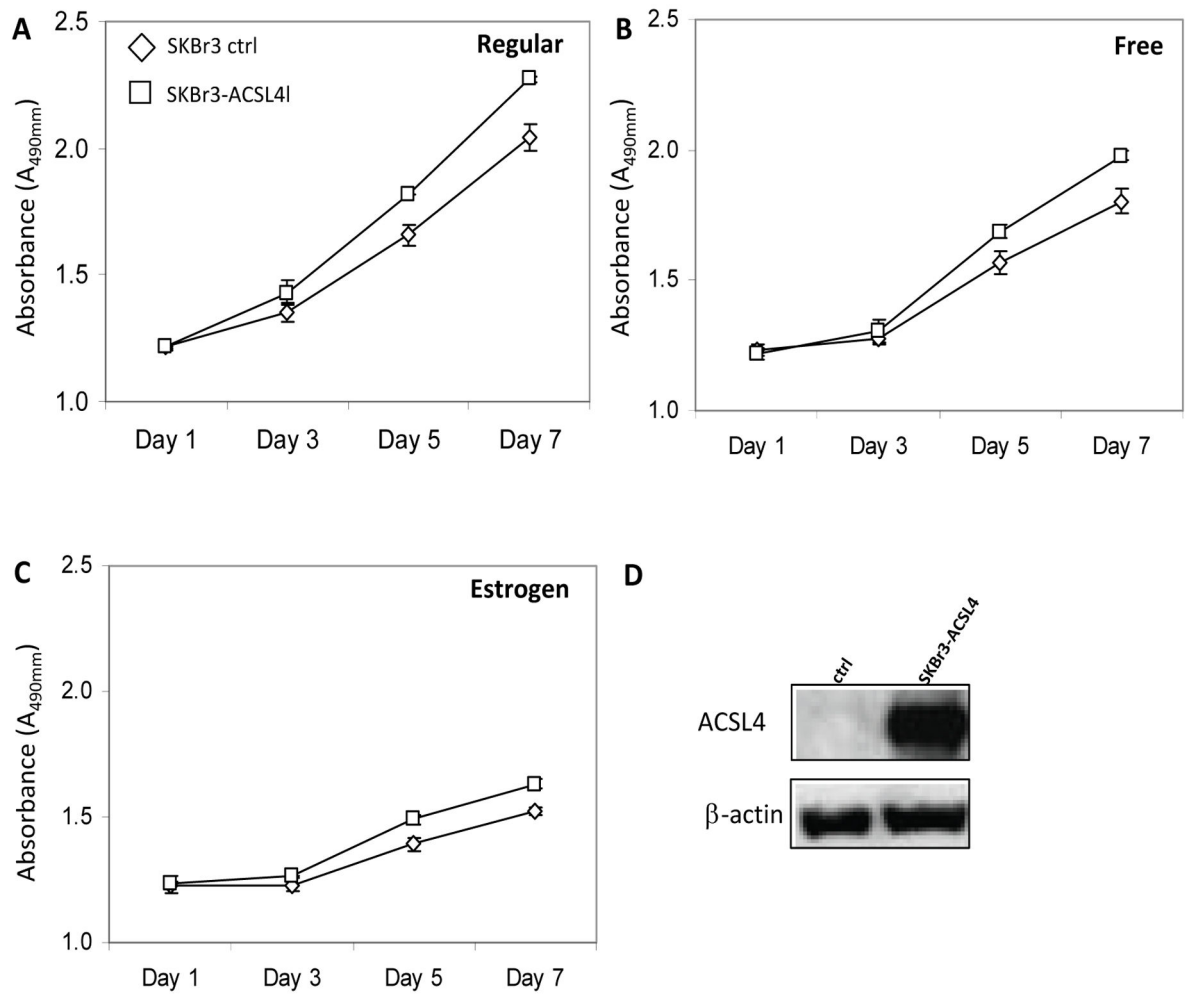
Effect of ACSL4 on growth of SKBr3 cells. Control- and ACSL4-tranfected SKBr3 cells were cultured in complete medium (**A**), or phenol red-free, charcoal-stripped medium without (**B**) or with (**C**) added estrogen. Values shown are the means of triplicate determinations ± 1SD. The differences between the curves are significant: **A**, p<0.0001; **B**, p<0.0001; **C**, p<0.0001. Panel **D**, immunoblot analysis of whole cell lysates for expression of ACSL4 in control and ACSL4-transfected cells.

 Data presented in table 1 indicates that ACSL4 expression is inversely correlated with that of HER2 overexpression. We examined whether simultaneous expression of HER2 and ACSL4 might impact cell growth in a manner similar to that seen for simultaneous expression of ER and ACSL4. To test this, the HER2-positive, ACSL4-negative cell line, SKBr3, was stably transfected with ACSL4 cDNA using a lentivirus vector, and the effects on growth monitored. Figure 7 illustrates that ACSL4 expression results in a modest increase in growth compared with control-transfected cells. The same effect was observed in medium containing complete (7A) as well as charcoal-stripped serum, with (7C) or without (7B) added estrogen. 

### ACSL4 expression impacts sensitivity to targeted as well as traditional treatment reagents

**Figure 8:**
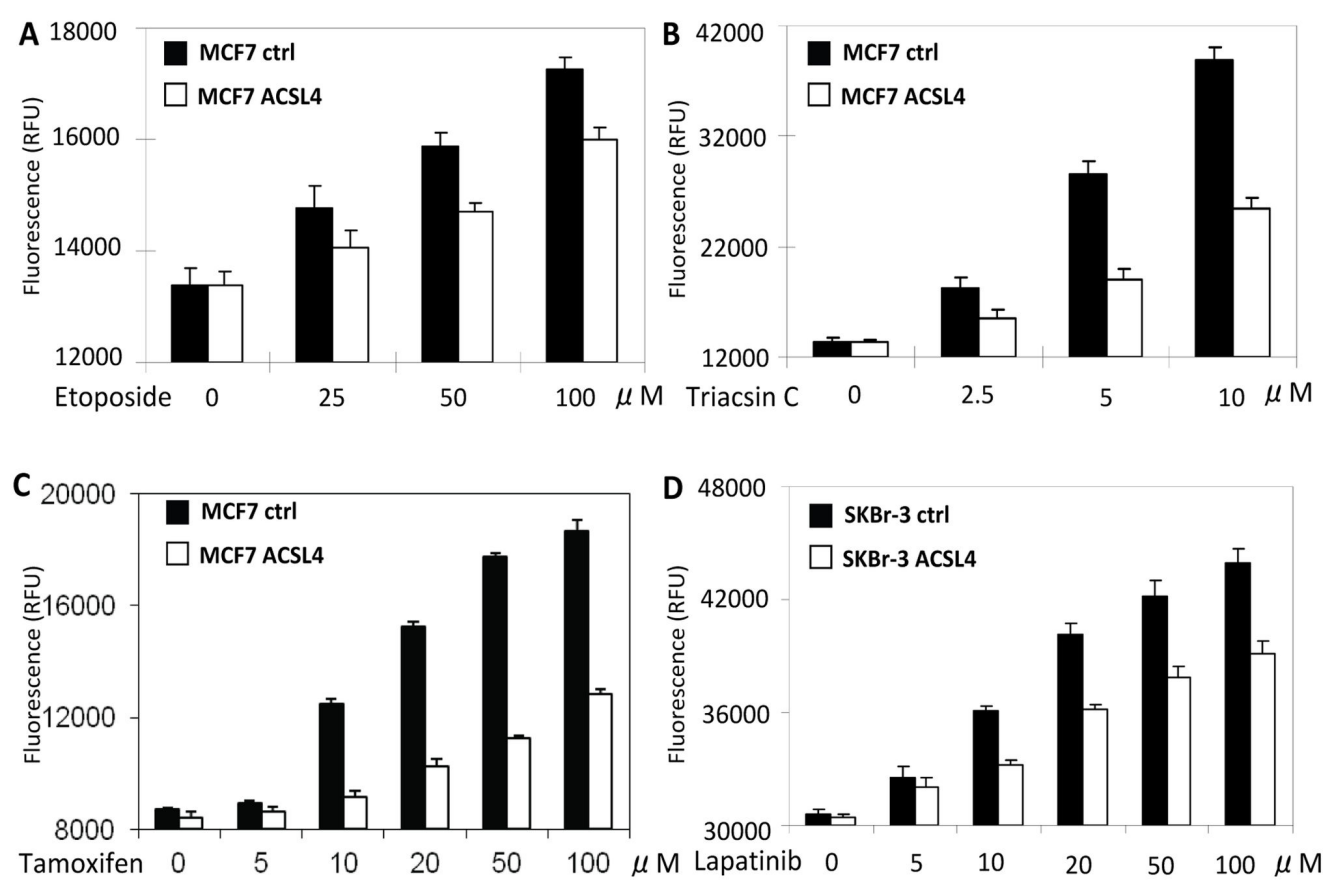
Effect of ACSL4 expression on apoptosis in MCF-7 and SKBr3 cells. Control and ACSL4-transfected MCF-7 cells were treated with varying doses of etoposide (**A**), triacsin C (**B**) or tamoxifen (**C**) for 48 hours and apoptosis measured as described in the text. For Panel **D**, control and ACSL4-transfected SKBr3 cells were treated with lapatinib for 48 hours and apoptosis measured as described in the text. Results shown are the means of triplicate determinations ± 1SD. Significance of the differences are **A**, p=0.027; **B**, p=0.012; **C**, p=0.005, **D**, p=0.008.

The loss of estrogen’s stimulatory effect as a result of ACSL4 expression as shown above suggested that ACSL4-expressing MCF-7 cells might be less sensitive to tamoxifen treatment than control cells. To test this hypothesis, the response of control and ACSL4-MCF-7 cells to various treatments was compared. Figure 8 (A-C) documents the differential effects of tamoxifen, etoposide and the ACSL inhibitor, triacsin C, on control- and ACSL4-MCF-7 cells. In each case, expression of ACSL4 correlates with increased resistance to the therapeutic regimen, suggesting an overall effect to increase survival in general. These findings are in line with those previously reported suggesting that ACSLs, in general, are cancer survival factors that can inhibit the efficacy of etoposide [32]

SKBr3 cells are normally sensitive to treatment with reagents that target HER2 activity. We tested the effect of ACSL4 expression on sensitivity to one of these reagents, lapatinib. Figure 8D demonstrates that ACSL4 expression induces some resistance to lapatinib as evidenced by decreased apoptosis in the lapatinib-treated SKBr3 cells that express ACSL4. 

### Impact of ACSL4 expression on down stream mRNA and protein expression

**Figure 9:**
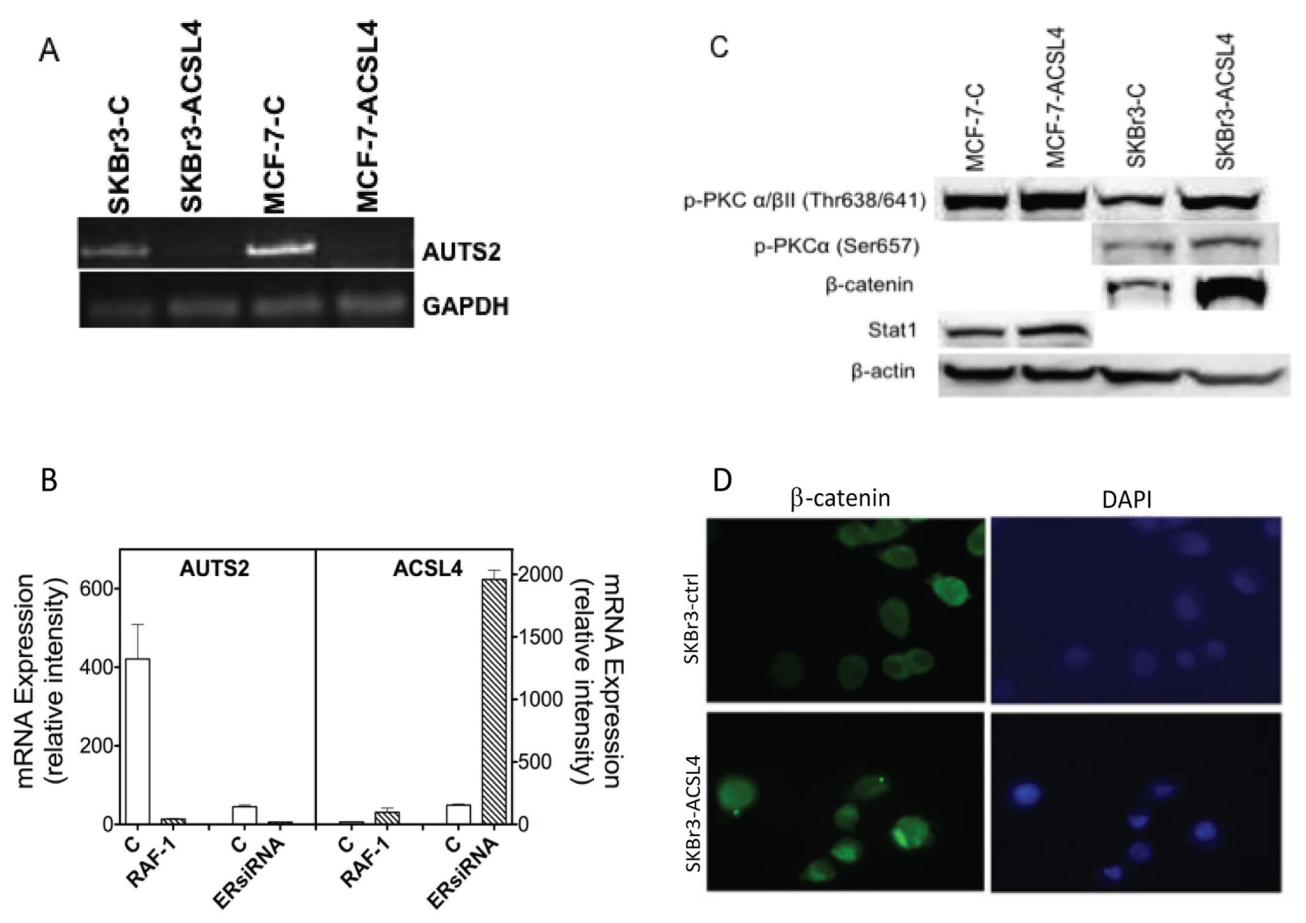
Validation of microarray and proteomic pathway analysis data. Panel **A** illustrates results from a semi-quantitative RT-PCR analysis of control- and ACSL4-transfected MCF-7 and SKBr3 cells. Both transfections were carried out with lentivirus vectors as described in the text. Panel B documents changes in AUTS2 expression as a result of transfection of MCF-7 cells with either RAF-1 or siRNA directed against ER. Values shown represent the means of three determinations ± 1SD. The differences between controls and transfected cells are significant, p=1.0E-03 and p=9.0E-06, respectively. Data were taken from microarray studies GDS1925 and GDS4061 deposited at http://www.ncbi.nlm.nih.gov/geo/ . Panel **C** depicts an immunoblot analysis of selected pathway proteins. Fold changes observed for expression of p-PKCα/βII (Thr638/641) are 1.4 in MCF-7 cells and 1.9 in SKBr3 cells; for expression of p-PKCα is 2.0 in SKBr3 cells; for expression of β-catenin is 35 in SKBr3 cells; and for expression of stat1 is 1.1 in MCF-7 cells. Panel **D** shows immunofluorescent staining of β-catenin in control and ACSL4-transfected SKBr3 cells. Methods were as described in the text.

We next evaluated gene expression as a function of ACSL4 expression using both integrated Affymetric microarray analysis and proteomic pathway array analysis (PPAA) comparing control and ACSL4-transfected MCF-7 and SKBr3 cells. For the Affymetric microarray analysis, we assessed 1) the impact of acute, conditional expression of ACSL4 in MCF-7 cells using a tetracycline inducible model as well as 2) the impact of long-term ACSL4 expression in MCF-7 cells stably expressing ACSL4; and 3) the impact of long-term ACSL4 expression in SKBr3 cells stably expressing ACSL4. Note that the mRNA expression values for ACSL4 in the transfected cells are not increased due to the fact that the probe is directed against an untranslated region of the mRNA that was not present in the transfected cDNA. Table S1 is a list of genes affected by ACSL4 expression that were common across experiments. Tables 6, 7 and 8 list those genes whose expression was increased or decreased by at least 2-fold. Only one gene satisfied these criteria across all three experiments (table 6), autism susceptibility candidate 2 (AUTS2). Additional common genes were observed when comparing acute and stable ACSL4 expression in MCF-7 cells (table 7) and between MCF-7 and SKBr3 cells (table 8). Figure 9A validates the microarray data with respect to AUTS2 mRNA expression in both MCF-7 and SKBr3 cells. The precise function of AUTS2 is unknown; however mutations in this gene have been associated with neurodevelopmental disorders [33], as have mutations in ACSL4, which have been implicated in both X-linked metal retardation [34] and autism [35]. In order to further analyze the inverse relationship between AUTS2 and ACSL4, we assessed the effect of inducing ACSL4 expression in MCF-7 cells by an alternate route. We have previously reported that constitutive expression of RAF-1 in MCF-7 cells causes induction of ACSL4 mRNA [7], and in the present study we report that ACSL4 is induced in MCF-7 cells as a result of siRNA-induced ablation of ER (Figure 5). Figure 9B shows the effect of these manipulations on AUTS2 mRNA expression. In both cases AUTS2 mRNA expression is decreased by 90% and 88% respectively. 

**Table 6:** Shared affected genes: Comparison across all three experiments.

Probe Set ID	**Gene Symbol**	**FC MCF-7-1^1^**	**p-value**	**FC MCF-7-2^2^**	**p-value**	**FC SKBr3**	**p-value**
212599_at	**AUTS2**	**-2.48**	**7.16E-04**	**-2.50**	**7.71E-03**	**-2.41**	**3.04E-04**

Microarray data generated as described in the text. Full results can be found in table S1.

1 Compares conditional, doxycycline-induced expression of ACSL4 in MCF-7 cells with doxycycline-treated control-transfected cells.

2 Compares control vector and ACSL4-transfected MCF-7 cells

FC = fold change

**Table 7:** Shared affected genes: Comparison of conditional and stable induction of ACSL4 in MCF-7 cells.

Probe Set ID	**Gene Symbol**	**FC MCF-7-1^1^**	**p value**	**FC MCF-7-2^2^**	**p value**
212599_at	**AUTS2**	**-2.48**	**7.16E-04**	**-2.50**	**7.71E-03**
207886_s_at	**CALCR**	**-3.11**	**3.61E-03**	**-2.13**	**1.79E-03**
224994_at	**CAMK2D**	**-2.87**	**5.45E-04**	**-4.18**	**4.30E-03**
209479_at	**CCDC28A**	**2.03**	**1.29E-05**	**2.07**	**2.81E-02**
231766_s_at	**COL12A1**	**-5.22**	**1.04E-03**	**-3.44**	**6.23E-04**
224822_at	**DLC1**	**-2.18**	**5.02E-05**	**-2.31**	**6.47E-03**
230263_s_at	**DOCK5**	**-2.01**	**4.45E-03**	**-2.11**	**2.18E-04**
1555606_a_at	**GDPD1**	**4.77**	**7.83E-04**	**2.68**	**3.86E-02**
214469_at	**HIST1H2AE**	**6.03**	**6.24E-03**	**2.05**	**4.72E-02**
205842_s_at	**JAK2**	**-2.69**	**1.60E-02**	**-2.04**	**3.89E-02**
201505_at	**LAMB1**	**-3.99**	**1.01E-02**	**-2.29**	**1.23E-02**
227761_at	**MYO5A**	**-2.70**	**1.16E-03**	**-2.32**	**1.07E-02**
213988_s_at	**SAT1**	**2.18**	**4.30E-05**	**4.06**	**1.28E-02**
226051_at	**SELM**	**2.03**	**9.82E-03**	**2.63**	**4.78E-03**
210664_s_at	**TFPI**	**2.20**	**2.32E-03**	**2.80**	**3.05E-02**
203887_s_at	**THBD**	**-2.38**	**3.17E-04**	**-2.16**	**2.27E-02**
227671_at	**XIST**	**-12.40**	**3.05E-07**	**-77.05**	**1.36E-02**

Microarray data generated as described in the text. Full results can be found in table S1.

1 Compares conditional, doxycycline-induced expression of ACSL4 in MCF-7 cells with doxycycline-treated control-transfected cells.

2 Compares control vector and ACSL4-transfected MCF-7 cells

FC = fold change

**Table 8:** Shared affected genes: Comparison of stable induction of ACSL4 in MCF-7 and SKBr3 cells.

Probe Set ID	**Gene Symbol**	**FC MCF-7-2**	**p value**	**FC SKBr3**	**p value**
212599_at	**AUTS2**	**-2.50**	**7.71E-03**	**-2.41**	**3.04E-04**
235626_at	**CAMK1D**	**-2.84**	**6.88E-05**	**-2.00**	**2.80E-05**
236313_at	**CDKN2B**	**-2.74**	**4.31E-04**	**-2.26**	**7.46E-05**
229088_at	**ENPP1**	**2.08**	**4.82E-04**	**2.53**	**1.57E-04**
218796_at	**FERMT1**	**-2.01**	**1.92E-03**	**-3.09**	**2.01E-03**
203710_at	**ITPR1**	**-3.17**	**1.20E-02**	**-2.68**	**1.51E-05**
203939_at	**NT5E**	**-3.81**	**2.20E-04**	**-4.86**	**3.70E-04**
208510_s_at	**PPARG**	**2.34**	**1.96E-02**	**2.17**	**1.56E-02**
228396_at	**PRKG1**	**-2.21**	**9.74E-03**	**-4.04**	**1.60E-02**
223168_at	**RHOU**	**-2.35**	**3.64E-02**	**-3.18**	**9.16E-07**

Microarray data generated as described in the text. Full results can be found in table S1.

FC = fold change

An analysis of pathways common to all three experiments (table S2) indicates that ACSL4 expression results in the down regulation of a number of signal transduction pathways, including those involving both steroid and peptide hormones and growth factors, which supports the notion that ACSL4 expression induces hormone/growth factor resistance. In addition, pathways involving cytoskeletal organization and cell adhesion are also down regulated, as might be expected in light of the observed effect of ACSL4 expression on invasion. Also consistent with a more aggressive phenotype is a reduction in the expression of genes involved in apoptosis. The ability of ACSL4 to increase proliferation is reflected in the upregulation of pathways that function in general cellular metabolism and DNA and RNA synthesis. 

Results from PPAA analysis comparing control- and ACSL4-expressing MCF-7 and SKBr3 cells are shown in table 9. The pathways that appear activated by expression of ACSL4 in both cell lines are those involving PKCα/βII (Thr 638/641), L-selectin and alcohol dehydrogenase (ADH). In SKBr3 cells β-catenin levels were greatly increased. Figure 9C validates the increases seen in phospho-PKCα and β-catenin suggested by the PPAA results. The increase in β-catenin in SKBr3 cells is concomitant with an increased nuclear localization, as illustrated by the immunofluorescense study shown in Figure 9D. A role for the WNT signaling pathway in breast cancer progression has been suggested by data illustrating that loss of the WNT negative regulator, sFRP1, is associated with breast cancer progression and poor prognosis [36]. When ACSL4-positive MDA-MB-231 cells are forced to express sFRP1, ACSL4 mRNA expression decreases by 42% (p = 0.003), as illustrated in microarray data reported by Matsuda et al [37] . This data is deposited on the GEO website, GSE13806.

**Table 9:** PPAA analysis of pathway protein expression as a function of ACSL4 expression.

	**MCF-7 Cells**		**SKBr3**	
	Fold Change*		**Fold Change**	
	**Exp. 1**	**Exp. 2**	**Exp. 1**	**Exp. 2**
**Protein**				
p-PKCα (Ser657)	NC	NC	1.59	1.83
p-PKC α/βII (Thr638/641)	1.66	2.04	2.16	1.78
Stat1	1.22	2.14	NC	NC
cdc25B	0.48	0.83	NC	NC
EGFR	1.09	2.32	NC	NC
Hsp90	NC	NC	0.35	0.74
PCNA	NC	NC	0.38	0.78
p38β	NC	NC	0.30	0.67
β-catenin	NE	NE	20.93	16.83
XIAP	0.51	0.52	NC	NC
OPN	NE	NE	1.32	7.53
WT1	NC	NC	0.47	0.76
NFkBp50	NC	NC	0.57	0.49
Calretinin	0.82	0.14	NC	NC
ICAM-1	0.37	0.26	NC	NC
c-Flip	NC	NC	0.36	0.53
Rab 7	1.54	1.82	2.53	2.18
Bak	1.92	2.03	NC	NC
Nkx-3.1	NC	NC	1.72	1.76
RIP	0.48	0.66	NC	NC
ERCC1	NC	NC	2.77	1.38
L-Selectin	2.28	3.01	2.48	1.45
Cytokeratin 18	NC	NC	0.55	0.47
FAH	NC	NC	3.31	1.49
LSD1	NC	NC	2.23	1.62
LKB1	NC	NC	0.77	0.43
PEDF	NC	NC	0.41	0.10
SPAK	NC	NC	0.45	0.04
ADH	0.52	0.23	0.69	0.43

Methods were as described in the text and results shown are from two separate experiments. Fold change = ACSL4-transfected cells/control cells NC = no change; NE = not expressed 

## Discussion

 Breast cancer is a heterogeneous disease in which treatment is complicated by varying degrees of steroid hormone/HER2 sensitivity and the development of resistance to therapies targeting the actions of these hormones and growth factors. The ability to characterize individual cancers as to their likelihood of responding to such therapies currently relies on the measurement of steroid hormone/HER2 receptors, their presence being indicative of a probable response to a therapy designed to block their action. However, *de novo* and acquired resistance to targeted therapies remains a frequent problem in hormone receptor-expressing as well as Her2 positive breast cancers [38,39]. We have presented evidence here that expression of a lipid metabolic enzyme, ACSL4, inversely correlates with the presence of steroid hormone and growth factor receptors in breast cancer, and as such may be a marker for the highly aggressive QNBC. The addition of AR to the triple negative characterization is supported by a variety of reports suggesting that TNBC that lack AR have a worse prognosis than AR-positive TNBC [5,40-44]. This inverse correlation between ACSL4 status and receptor status was significant for both studies in cell lines as well as tumor samples. 

 In order to determine whether ACSL4 status could serve as a biomarker for QNBC, we surveyed ACSL4 expression in 71 different cell lines and correlated the data with that for receptor expression. ACSL4 status predicted QNBC status with a sensitivity of 78% and a specificity of 86% (table 4). Of potential interest are those instances where either both ACSL4 and receptors are co-expressed or neither ACSL4 nor receptors are expressed (table 5). These data suggest the possibility of a further stratification of both receptor-positive breast cancer and QNBC based on ACSL4 status that might predict prognosis and/or response to therapy. 

Stratifications have also been proposed based on intrinsic molecular subtype as defined by a variety of gene expression paradigms [29,45]. ACSL4 status is clearly associated with claudin-low, and to a lesser extent, basal-like breast cancer (Figure 1). There does not appear to be an association between ACSL4 status with respect to luminal subtypes [46]. This brings up the question of why ACSL4 has not been identified in any previous gene signature of molecular subtypes. We would suggest the possibility that the low level of relative expression values seen in positive samples as compared with negative samples underestimates the actual change observed in ACSL4 expression as a function of molecular subtype and receptor status, and thus ACSL4 is rejected as a function of the application of algorithms designed to capture the most significant differences. However, immunoblot data confirms that a log base 2 value of 3.5 can represent strong expression while a value of 2.5 is negative [7]. 

In support of ACSL4 functioning as a biomarker for effective QNBC, the data detailed here indicate that forced ACSL4 expression is capable of inducing resistance to hormone-stimulated growth as well as reducing sensitivity to targeted therapies. In estrogen sensitive MCF-7 cells, expression of ACSL4 confers insensitivity to estrogen treatment with respect to growth as well as increased invasion capability (figures 2-4). In fact, there is a slight inhibition of growth in the presence of estrogen. These effects are accompanied by a reduction in expression of ER, PR and AR (Figure 2). ACSL4 expression in the absence of any hormonal treatment increases the growth rate of the cells in both complete serum (data not shown) as well as in charcoal-stripped serum. Similar results have been recently reported for ACSL4-expressing MCF-7 cells both *in vitro* and *in vivo* [47]. Reduction of ACSL4 levels in ACSL4-positive, QNBC, and MDA-MB-231 cells significantly inhibits invasion capability (Figure 4), confirming previous data from Maloberti et al [12]. Lastly, ACSL4 expression decreases apoptosis in MCF-7 cells in response to treatment with tamoxifen (ER antagonist), triacsin C (ACSL1, 3 and 4 antagonist) or etoposide (Figure 8). Thus the ability of ACSL4 to enhance survival is not limited to effects on estrogen action, but appears also to have a general overall effect. We have previously reported that attenuation of ACSL4 expression in the QNBC cell line, MDA-MB-231, causes increased sensitivity to treatment with triacsin C [7]. ACSL5 has previously been reported to function as a cancer survival factor [32,48]

Not only is ACSL4 expression able to reduce ER expression, but an siRNA-mediated reduction of ER in MCF-7 cells induces expression of ACSL4 as shown in Figure 5. Results shown in Figure 5 were derived from microarray data reported by Al-Saleh et al [31]. Lastly, the proliferative effect of ACSL4 is also observed in a xenograft mouse model utilizing both intact and ovariectomized nude mice (Figure 6). Since nude mice have low levels of endogenous estrogen, and the intact mice were not treated with exogenous estrogen, it is not surprising that there is little difference observed between intact and ovariectomized mice. It will be interesting to see whether estrogen supplementation in the ovariectomized model inhibits growth of ACSL4-transfected MCF-7 cells, as is the case *in vitro.*

In the HER2-overexpressing cell line, SKBr3, stable expression of ACSL4 results in a slight increase in the rate of growth (Figure 7), as well as reduced sensitivity to treatment with lapatinib (Figure 8D), supporting the hypothesis that simultaneous expression and overexpression of both ACSL4 and HER2, respectively, are indicative of resistance to HER2-based therapy. Estrogen appeared to inhibit growth in the experiment shown in Figure 7. Further experimentation will be required to determine the significance of this inhibition in the absence of ER expression.

 The effect of ACSL4 expression on overall gene expression was evaluated by microarray studies in two different cell lines: a conditional and stable MCF-7 model, as well as a stable SKBr3 model. Table S1 details the common affected genes across the three models, and Table S2 lists common affected pathways. Table 6 lists those shared affected genes across the three models that were decreased by ≥50% or increased by ≥100%. The only gene whose expression was so affected was AUTS2, an effect validated by RT-PCR (Figure 9A). The function of this gene is thought to be associated with neural development, and mutations in AUTS2 have been linked to autism [35,49], although the exact nature of its activity remains unknown. The prevalence of autism in males suggests involvement of the X chromosome, and the association between X-linked mental retardation, a syndrome associated with mutations in ACSL4, which is located on the X-chromosome [34], and autism support the finding reported here that ACSL4 expression regulates AUTS2 expression. 

The ability of ACSL4 to cause a decrease in X inactive specific transcript (XIST) in both MCF-7 models is of interest in light of the data implicating a possible role for this RNA moiety in the genesis of breast cancer [50]. Like ACSL4, XIST is located on the X chromosome. 

When common affected pathways are analyzed, it becomes clear that expression of ACSL4 causes a general increase in cellular metabolism, including RNA and DNA synthesis, as well as a decrease the expression of pathways involved in signal transduction and cytoskeletal organization.

Table 9 lists changes in pathway-associated protein expression or phosphorylation state as a function of ACSL4 expression in MCF-7 and SKBr3 cells. Changes in p-PKCα, and β-catenin were validated (Figure 9C). The increase in β-catenin in SKBr3 cells was also verified by immunofluorescence, and it was noted that there was increased nuclear localization as a result of ACSL4 expression (Figure 9D).

Recent studies have addressed the mechanism by which ACSL4 induces an aggressive phenotype in breast cancer [12,47]. ACSL4 is an enzyme in the lipogenic pathway that supplies activated fatty acids for use in glycerolipid synthesis and in β-oxidation. There is even some evidence that activated fatty acids (fatty acyl-CoAs) function as transcription factors [51]. The preference of this enzyme for arachidonic acid as a substrate has lead to the hypothesis that ACSL4 expression decreases the availability of free arachidonic acid for conversion to leukotrienes and eicosanoids, and thus functions to impede the production of these compounds, a suggestion that has been supported by data [13,52]. In the case of breast cancer, however, ACSL4 has been demonstrated to increase production of lipoxygenase products, an effect attributed to increased uptake of AA-CoA into the mitochondria, followed by regeneration of free AA for subsequent conversion to prostaglandins [12]. ACSL4 expression also induces increased expression of COX-2. Thus ACSL4 activity increases the pool of free AA available for conversion to prostaglandins, as well as increasing enzyme activity (COX-2) involved in prostaglandin synthesis. The result is a more aggressive breast cancer phenotype. In prostate cancer, induction of COX-2 has been shown to be effected by addition of AA *via* the PI3K/AKT pathway [53]. Whether or not this effect involves ACSL4 activity remains to be determined.

The unique subcellular localization of ACSL4 to peroxizomes and mitochondria-associated endoplasmic reticulum membranes suggests a possible role for this enzyme in facilitating oxidation of free fatty acids as a source of energy in proliferating tumor cells. Thus both substrate specificity and subcellular localization are currently under investigation in our laboratory in order to determine their role in mediating the effects of ACSL4 expression on breast cancer phenotype. 

Breast cancer is not the only cancer demonstrated to differentially express ACSL4. It has previously been reported that ACSL4 expression is associated with the malignant phenotype in both liver and colon cancer and functions to modulate proliferation [10,11]. In breast cancer, this association is limited to the most aggressive forms of the disease, with the more benign, receptor positive cancers being negative for ACSL4 expression. Since normal mammary epithelium is predominantly receptor-negative, ACSL4 expression is positive for normal compared with most (i.e. receptor-positive) breast cancer tissue. The precise role of ACSL4 activity in generating the malignant phenotype seen in colon, liver and aggressive breast cancers remains to be determined. Its utility as a biomarker for differentiation of breast cancer molecular subtypes and for prediction of treatment response and prognosis is supported by the evidence presented here and in prior studies by our group and others. Further work will be required to demonstrate that routine measurement of ACSL4 in breast tumor samples might obviate the need for hormone/HER2 receptor measurements in cases where ACSL4 is positive, or be useful as a marker, in addition to receptor status, in making treatment decisions. Of particular interest is a possible role for ACSL4 as a mediator of the racial disparity observed in the increased prevalence of TNBC in the African American population. Microarray analyses performed on liver tissue from patients undergoing weight loss surgery revealed elevated levels of ACSL4 mRNA in African Americans when compared to Caucasians [54]. 

## Conclusions

An analysis of the expression of ACSL4 in breast cancer indicates that there is an inverse relationship between the presence of this lipid metabolic enzyme and the steroid hormone/HER2 receptor status of the sample. In a study of 71 different breast cancer cell lines, ACSL4 status predicted QNBC status with a sensitivity of 78% and a specificity of 86%. Higher ACSL4 expression was also associated with TNBC tumor samples. In cases where ACSL4 expression status failed to predict receptor status, it is possible that a sub classification based on ACSL4 status might have prognostic or therapeutic implications. Including ACSL4 expression data in gene signatures designed to assess intrinsic molecular subtype might improve the accuracy of these determinations. There is also the possibility that adding ACSL4 measurements to current measurements of sex steroid and HER2 receptors would increase the predictive value of current protocols. Studies assessing the effect of ACSL4 expression in breast cancer cell lines suggest that ACSL4 functions as a mediator of hormone independence and resistance to hormonal and chemotherapy. Thus simultaneous expression of ACSL4 and a receptor might serve as an indicator of resistance to targeted therapies, while it is possible that receptor-negative tumors that are also ACSL4-negative might be less aggressive than ACSL4-positive tumors and be amenable to receptor-based therapies. In essence, ACSL4 status may be a biomarker that further defines receptor status. Given that receptor status does not always predict intrinsic molecular subtype, inclusion of ACSL4 status as a biomarker might prove useful as a prognostic and therapeutic guide. In addition, ACSL4 itself might serve as a useful target for development of future therapies.

## Supporting Information

Table S1 **Common affected genes.** Results from gene expression studies comparing control-transfected and ACSL4-transfected MCF-7 and SKBr3 cells. Methods were as described in the text. MCF-7 cells were transfected with either a doxycycline-inducible expression vector (MCF-7-1) or a lentiviral expression vector (MCF-7-2). SKBr3 cells were transfected with a lentiviral vector.
(XLS)

Table S2 **Common affected pathways.** An analysis of the results detailed in table S1 using methods described in the text.
(XLS)
